# Possible dissolution mechanism of alkali lignin in lactic acid-choline chloride under mild conditions†

**DOI:** 10.1039/d0ra07808e

**Published:** 2020-11-09

**Authors:** Zhuang Liu, Yi Hou, Songqing Hu, Youming Li

**Affiliations:** State Key Laboratory of Pulp and Paper Engineering, South China University of Technology Guangzhou 510640 P. R. China ceyhou@scut.edu.cn; School of Food Science and Engineering, South China University of Technology Guangzhou 510640 P. R. China

## Abstract

In this study, several representative deep eutectic solvents (DESs) were designed to evaluate the solubility for alkali lignin (AL). It was found that DESs with lactic acid (LA) as hydrogen bond donors (HBDs) had good solubility for AL, in which lactic acid(LA) : choline chloride(ChCl) 10 : 1 showed excellent solubility with more than 17 wt% under a relatively mild condition of 60 °C. The results of gel permeation chromatography (GPC), FTIR and ^1^H and HSQC NMR spectroscopies revealed an important possible dissolution mechanism of AL in LA-ChCl, that is, AL could be depolymerized under the action of LA when dissolved in LA-ChCl. Then, a new connection would form between the phenolic groups on the lignin fragments and ChCl, which is similar to that between ChCl and LA in DES, leading to an increase in the molecule weight of lignin. The new connections could be easily broken under the action of heat (150 °C) or microwave to the redispersion of lignin fragments. The results would provide a theoretic base for the high-value application of lignin in bioresources.

## Abstract 2

In this study, several representative deep eutectic solvents (DESs) were designed to evaluate the solubility for alkali lignin (AL).

## Graphical abstract

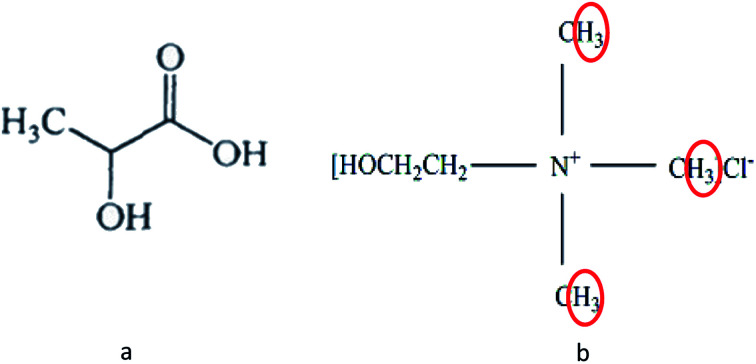

## Introduction

Lignin, one of the three main components of lignocellulose, has continuously generated increasing interests.^1^ Despite its great potential to produce a wide range of chemicals, lignin remains severely underutilized because of weak solubility in most existing solvents.^2^ Therefore, it is critical to develop effective solvent systems for lignin dissolution. Several solvent systems such as ionic liquids (ILs)^3–5^ and organic solvent^6^ have been designed and used; however, some drawbacks, such as high cost, high viscosity and complex synthesis process of ILs, environmentally unfriendly and toxicity of organic solvents, still restrict the practical applications of these solvent systems.

As an alternative to ionic liquids, deep eutectic solvents (DESs), which are composed of two or more hydrogen bond donors (HBDs) and hydrogen bond acceptors (HBAs), were designed by Abbott *et al.*^7^ The key properties of DESs are their low vapour pressure, high tunability and biodegradability based on their starting constituents. In addition, DESs can be prepared by simply heating a suitable HBD and HBA mixture, thus avoiding the use of expensive and toxic reagents and organic solvents in the preparation of ILs. Therefore, DESs have attracted increasing attention in various fields such as electrochemistry, separation, catalysis and extraction.^8–10^

In recent years, increasing attention has been paid to the application of DESs in biomass pretreatment;^11–14^ however, there are few studies on the application and mechanism of DESs in lignin dissolution.^15^ Maria *et al.*^16^ tested the solubility of lignin in more than 25 DESs, but none of them could be more than 15 wt%. J. G. Lynam *et al.*^17^ tested the solubility of AL in 5 DESs at 60 °C with the best solubility of 14 wt%. Therefore, it is necessary to find out a good DES suitable for alkali lignin and clarify its dissolution mechanism.

In this study, several representative deep eutectic solvents (DESs) were designed, and the solubility of these solvents for alkali lignin was tested. A microscope was used to observe the real state of the alkali lignin dissolving in these DESs. The composition analysis of lignin was assessed *via* gel permeation chromatography (GPC), and the structural characteristics were fully investigated *via* Fourier transform infrared spectroscopy (FT-IR) and ^1^H and ^1^H–^13^C correlation two dimensional (2D) heteronuclear single-quantum coherence (HSQC) nuclear magnetic resonance (NMR).

## Experimental

### Materials and methods

#### Materials

Alkali lignin was obtained by the acid precipitation and purification of black liquor from softwood soda pulp.

#### Preparation of DESs

The DESs used in this work were prepared by simply heating mixtures of HBDs and HBAs with a desired molar ratio (Table 1). Specifically, HBAs and HBDs weighed at a certain molar ratio were put into a beaker and mixed. The mixtures were heated and stirred in a 60 °C water bath until the solutions became clear and transparent. Then, the configured DESs were placed into reagent bottles and stored at room temperature.

**Table 1:** List of DES families investigated and the respective molar ratio

HBD	HBA	Molar ratio
LA	ChCl	2 : 1
LA	ChCl	10 : 1
LA	Urea (U)	2 : 1
LA	U	10 : 1
Butanoic acid (BA)	ChCl	2 : 1
BA	ChCl	10 : 1
BA	U	2 : 1
BA	U	10 : 1

#### The solubility of alkali lignin

The dissolution of alkali lignin in the as-synthesized DESs was conducted through a process based on a reported route.^17^ First, the desired DES (2.0 g) was added into a glass flask of 25 mL at the desired temperature (60 °C). When the temperature was constant, the alkali lignin was added with a weight of 10 mg each time, at the same time, under the constant stirring of the mixture. After the added lignin was completely dissolved, the next addition followed. The above process was repeated until the lignin could not be dissolved in 12 h. The weight of each addition was recorded, and the solution was observed under a polarizing microscope to determine the real state of AL in the DES system. The solubility of lignin in different DESs at 60 °C could be calculated based on the following formula:
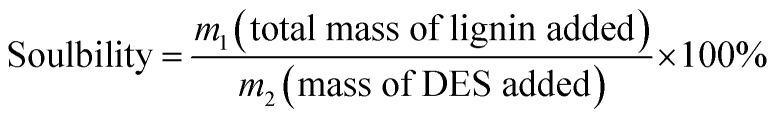


#### Dissolution of alkali lignin

DES (5 g) with good solubility in the previous step and AL (0.5 g) were added to a beaker. Then, the AL was dissolved in the chosen DES at 60 °C, 60 °C microwaves, 150 °C and 150 °C microwaves. The reaction time was recorded when alkali lignin dissolved completely.

#### Recovery of DES and DES lignin (DESL)

DESL was precipitated by adding an antisolvent (water : ethanol, 9 : 1) into the solution of DES. Then, the DESL and DES were separated by centrifugation (6000 rpm). The segregated lignin was freeze-dried, while the separated liquid could be recycled after removing the water and ethanol at a low temperature of 50 °C for 24–48 h.

### Characterization of AL and DESL

#### Gel permeation chromatography (GPC)

The molecular weight (*M*_w_) distribution of the lignin was evaluated by GPC using the acetylation of materials.^18,19^ Samples (0.3 g) were dissolved in 8 mL of pyridine–acetic anhydride (1 : 1, v/v), and the mixture was stirred in dark at room temperature for 72 h. Ethanol (1 mL) was then added to the mixture, and most of the pyridine–acetic anhydride was removed by a rotary evaporator. The dried samples were washed with water and collected on a 10 μm Nylon membrane filter. The acetylated materials were dissolved in tetrahydrofuran (THF) and analyzed by GPC using a Waters (USA) instrument under the following chromatographic conditions: column, Waters HR5E; column temperature, 40 °C; eluent, THF; flow rate, 0.22 mL min^−1^; detector, 2414 refractive index detector.

#### FTIR analysis

The chemical structures of various DESL were analyzed *via* Fourier transform infrared spectroscopy (Nexus Thermo Nicolet, USA). The samples were combined with KBr (1/50 mass ratio). Tests were taken in the range from 4000 to 400 cm^−1^ with a resolution of 4 cm^−1^.

#### ^1^H, 2D-HSQC NMR analysis

The DESL was characterized by ^1^H and 2D-HSQC NMR, and the NMR technologies were carried out on a Bruker AVANCE III HD 600 spectrometer (Bruker, Germany). Approximately 50 mg DESL was fully dissolved in 500 uL of dimethylsulfoxide-d6 (DMSO-d6). The mixture was then tested using the NMR spectrometer for 8 h. Matrices of 2048 data points for the ^1^H dimension from 160 to 0 ppm with a recycle delay of 1.5 s were collected. For the ^13^C-dimension, the spectral had recorded 128 increments of 64 scans, and matrices of 1024 data points were used. Bruker's Topspin 3.2 processing software was used for the semiquantitative analysis of the volume integrals of the NMR correlation peaks.

## Results and discussion

### The solubility of alkali lignin

**Fig. 1:**
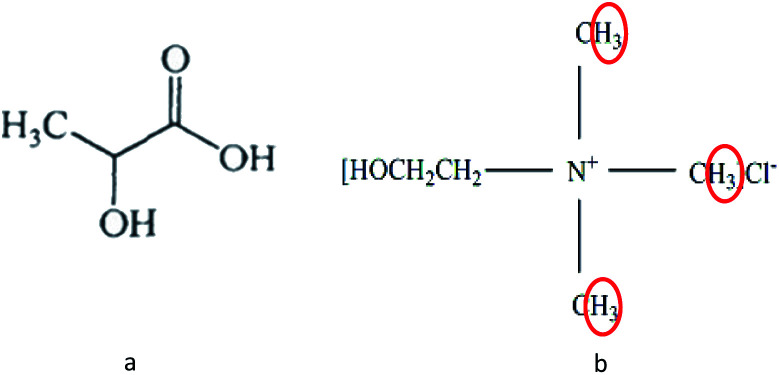
Structure of LA (a) and ChCl (b).

The solubility of alkali lignin in various DESs is shown in Table 2. It can be seen from Table 2 that the solubility of AL was excellent with LA as HBD in DESs. It may be due to the fact that LA itself contains hydroxyl groups (Fig. 1a), which would increase the polarity of the solution system.^20^ Therefore, the solubility of alkali lignin in LA itself was good (16.48%). However, a pulping trial with lactic acid alone, which aimed to treat wood chips, had been implemented with a prolonged time of 48 h compared to another trial of 8 h with ChCl-LA DES.^21^ Besides, the boiling point of LA : ChCl in the ratio of 10 : 1 DES (more than 150 °C) was higher than that of LA (122 °C), which would be safer to benefit the application. LA-based DESs would be more suitable than LA to be a solvent for dissolving lignin and biomass pretreatment.

**Table 2:** The solubility of alkali lignin in various DESs at 60 °C

DES	Solubility (wt%)
LA : U (2 : 1)	17.10
LA : U (10 : 1)	13.68
LA : ChCl (2 : 1)	11.80
LA : ChCl (10 : 1)	17.27
BA : U (2 : 1)	5.64
BA : U (10 : 1)	<1.00
BA : ChCl (2 : 1)	5.13
BA : ChCl (10 : 1)	<1.00
LA	16.48
BA	<1
Acetic acid : ChCl (2 : 1)^17^	12
LA : betaine (2 : 1)^17^	9
LA : proline (3.3 : 1)^17^	9

It can be seen from Table 2 that among the tested and reported DESs,^17^ LA : ChCl in the ratio of 10 : 1 was an excellent solvent for AL and a commonly used DES for biomass pretreatment.^12,22–24^ Therefore, LA-ChCl (LA : ChCl 10 : 1) was chosen as the DES to dissolve AL.

**Fig. 2:**
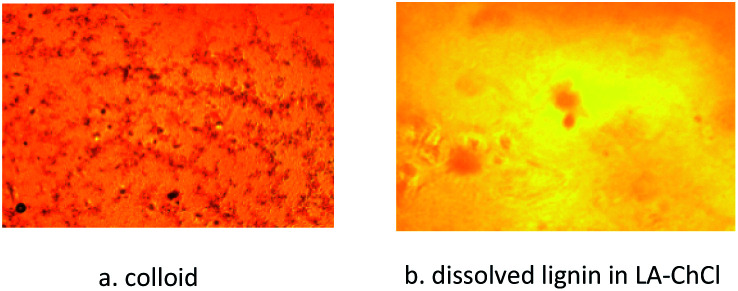
Colloidal and dissolved lignin in DESs under a microscope.

Due to the dark color and high viscosity of the mixture, it was difficult for the naked eye to identify whether it was a suspension or a solution. Therefore, a sample was observed under a polarizing microscope to determine the real state of AL in DES. Fig. 2 shows the actual state of the alkali lignin in LA-ChCl, where we can see that AL completely dissolved in LA-ChCl.

### Characterization and analysis of AL and various DESL

#### *M*_w_ distribution and analysis

**Fig. 3:**
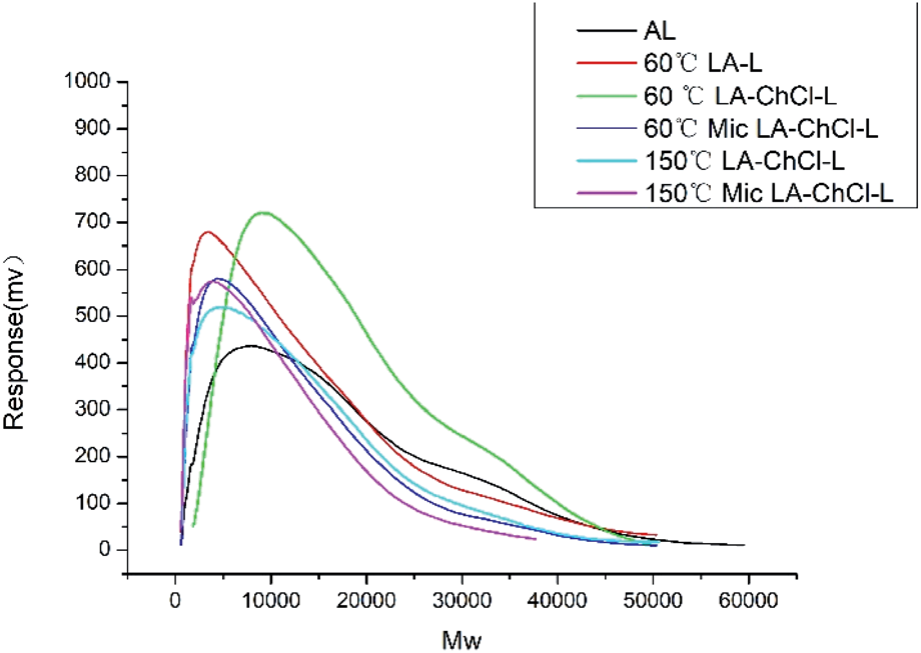
The *M*_w_ distribution of AL, 60 °C LA-L and lignin isolated from LA-ChCl on the condition of 60 °C, 60 °C with microwave, 150 °C and 150 °C with microwave.

In order to clarify the role of LA and ChCl for the dissolution of AL in LA-ChCl, AL was dissolved in LA and LA-ChCl at 60 °C. Also, to investigate the influence of temperature and microwave on the structure of dissolved lignin, AL was dissolved in LA-ChCl under the conditions of 60 °C with microwave, 150 °C and 150 °C with microwave, respectively. The lignin isolated from these treatments were named as 60 °C LA-L, 60 °C LA-ChCl-L, 60 °C Mic (Microwave) LA-ChCl-L, 150 °C LA-ChCl-L and 150 °C Mic LA-ChCl-L. The *M*_w_ distribution of this lignin was identified by the GPC analysis, which is shown in Fig. 3 and Table 3.

**Table 3:** The *M*_w_ distribution of various lignin samples

Sample	0–2000	2000–5000	5000–10 000	10 000+	*M*_n_	*M*_w_	PD
AL	11.50	25.29	26.90	35.70	4328	9819	2.2687
60 °C LA-L	23.60	31.80	23.00	21.60	2917	6879	2.3500
60 °C LA-ChCl-L	0.270	17.21	34.25	48.30	7780	12 053	1.5400
60 °C Mic LA-ChCl-L	19.69	32.62	26.09	21.61	3200	6842	2.1381
150 °C LA-ChCl-L	20.25	30.89	24.97	23.88	3233	7200	2.2270
150 °C Mic LA-ChCl-L	26.448	32.37	23.96	17.306	2710	5823	2.1487

It can be see from Table 3 and Fig. 3 that the *M*_w_ distribution in the range of above 10 000 and 5000–10 000 of 60 °C LA-L decreased compared with AL, and on the contrary, the *M*_w_ distribution in the range below 2000 and 2000–5000 increased significantly, resulting in the significant decrease in the average *M*_w_ for 60 °C LA-L. This result suggested that the AL was depolymerized under the action of LA.

It was interesting to find that the *M*_w_ distribution of 60 °C LA-ChCl-L in the range of 5000–10 000 and above increased significantly compared with 60 °C LA-L. On the contrary, the *M*_w_ distribution in the range below 2000 and 2000–5000 decreased, and the average *M*_w_ of 60 °C LA-ChCl-L increased. Besides, the polydispersity (PD) index values decreased clearly in 60 °C LA-ChCl-L, indicating more homogeneous molecular weight distribution in it.

Moreover, it is interesting to note that the *M*_w_ distribution of 60 °C Mic LA-ChCl-L and 150 °C LA-ChCl-L were found to be almost the same as that of 60 °C LA-L, and the average *M*_w_ of 150 °C Mic LA-ChCl-L was the smallest in the six researched lignin, indicating that the dissolution mechanism of AL in LA and LA-ChCl systems was different. ChCl played a special role in the dissolution process of AL in LA-ChCl, but this special effect was not stable and can be disintegrated by heating and external physical field.

#### FTIR analysis

**Fig. 4:**
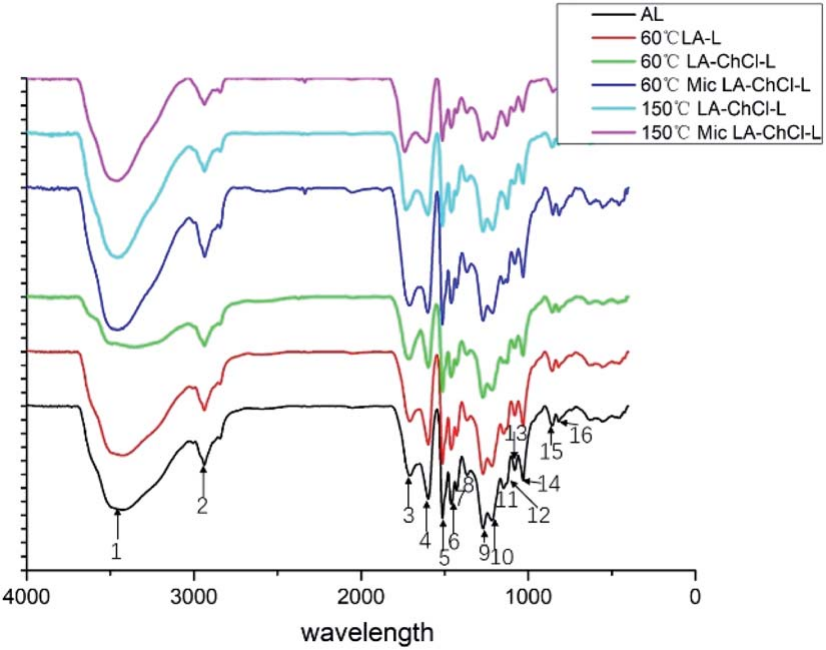
FTIR analysis of AL, 60 °C LA-L and lignin isolated from LA-ChCl on the condition of 60 °C, 60 °C with microwave, 150 °C and 150 °C with microwave.

Band assignments in the FTIR spectra of AL and various DESL were found based on previous reports.^25,26^ All lignin exhibited quite similar FT-IR spectra, which indicated the similarity of the main structure of the various lignin, as observed in Fig. 4.

The absorption band at around 1712 cm^−1^ mainly correspond to the C=O stretching vibrations in non-conjugated ketones, carbonyls, and ester of lignin groups. The typical signals at 1600 cm^−1^, 1513 cm^−1^ and 1429 cm^−1^, which represent that aromatic skeleton vibrations all appeared. This indicated that the aromatic skeleton had been kept well in the dissolution process. It was found that the peaks at 1148 cm^−1^, 1032 cm^−1^, 855 cm^−1^ and 819 cm^−1^ corresponded to the signals G-unit that appeared evidently in all lignin, indicating that the G-unit was relatively stable.

The peak at around 1259 cm^−1^, corresponding the C–O, C–O–C stretching vibration of lignin phenol and ether bond is mainly from the H-unit. The results suggested that all of the lignin showed the G- and H- units, and the signal of the S-unit did not show up, which was in line with the feature of softwood. The results showed that the main aromatic ring structure of lignin was effectively retained during the lignin dissolution process, which is conducive to the modification and high-value application of lignin in the later stage (Table 4).

**Table 4:** Band assignments in FTIR spectra of studied lignin

Number	Band cm^−1^	Assignment
1	3410	O–H stretching vibration in hydroxyl
2	2929	C–H stretching vibration in methyl and methylene
3	1712	C=O stretching (unconjugated)
4	1602	Aromatic skeletal vibration breathing with C=O stretching
5	1513	Aromatic skeletal vibration
6	1462	C–H deformation asymmetric
7	1429	Aromatic skeletal vibrations combined with C–H in-plane deformation
8	1367	Phenolic OH and aliphatic C–H in methyl groups
9	1259	C–O, C–O–C stretching vibration of lignin phenol and ether bond
10	1214	C–C plus C–O plus C=O stretch; G condensed > G etherified
11	1148	C–H in-plane deformation of G ring plus secondary alcohols plus C=O stretch
12	1126	Ether-O-
13	1081	C–O deformation in secondary alcohols and aliphatic esters
14	1032	Aromatic C–H in-plane deformation (G > S) plus C–O deformation in primary alcohols plus C=O stretch (unconjugated)
15	855	C–H out-of-plane in positions 2, 5 and 6 of G rings
16	819	C–H out-of-plane in positions 2, 5 and 6 of G rings

#### ^1^H and 2D-HSQC NMR spectra analysis

**Fig. 5:**
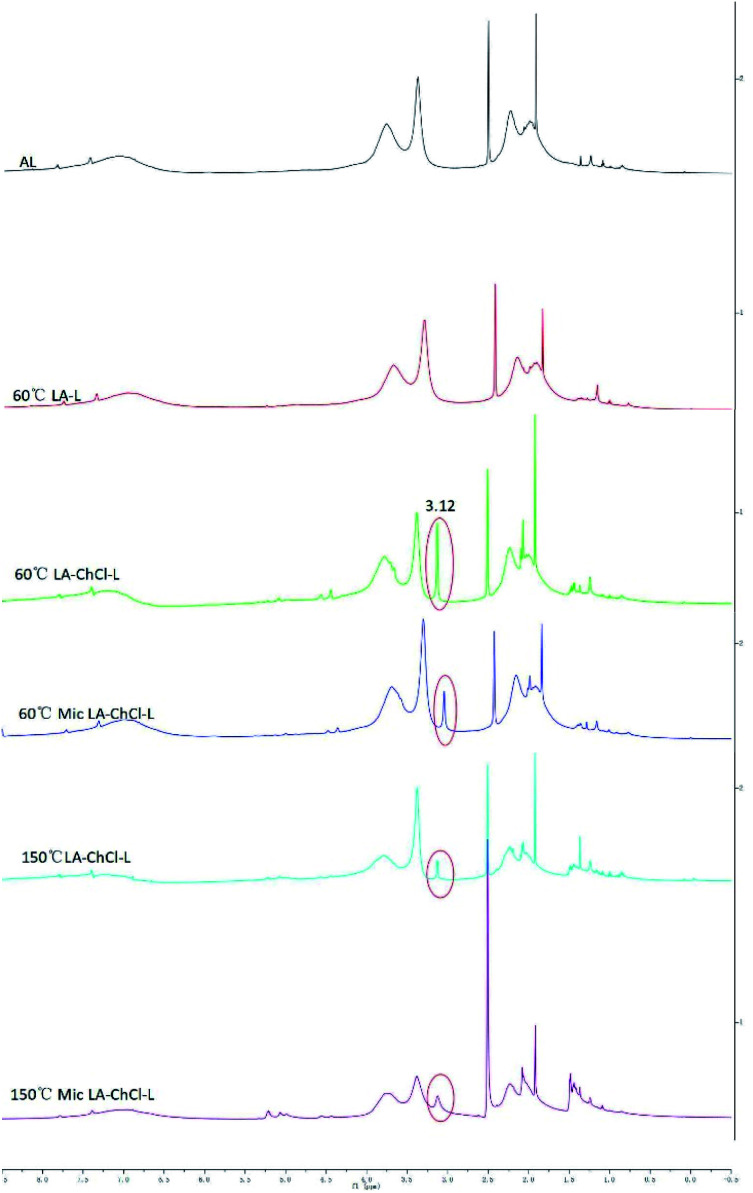
^1^H NMR spectra of AL, 60 °C LA-L and lignin isolated from LA-ChCl on the condition of 60 °C, 60 °C with microwave, 150 °C and 150 °C with microwave.

The AL, 60 °C LA-L, 60 °C LA-ChCl-L, 60 °C Mic LA-ChCl-L, 150 °C LA-ChCl-L and 150 °C Mic LA-ChCl-L were characterized by ^1^H and 2D-HSQC NMR techniques. The ^1^H NMR spectra of them are shown in Fig. 5, and the assignment of the main signals of them is shown in Table 5.

**Table 5:** Assignment of main signals in ^1^H NMR spectra of researched lignin

ppm	Proton type
0.10–3.61	Aliphatic side chain protons linked to benzene rings
0.79–0.84	Aliphatic protons
1.21	Protons on the alcohol hydroxyl group
2.50	DMSO-d6
3.12	Protons on the nitromethyl group of ChCl
3.51	Protons in CH_2_ of side chain
3.63–3.91	Methoxy protons
4.01–4.51	Hydrogen proton at γ site (Hγ) in the structure of β-O-4
4.18–4.23	Protons on the side chain (Cγ) of benzene rings
4.56–4.61	Hydrogen proton of α site (Hα) in β–β structure
4.62–4.91	Hydrogen proton of β site (Hβ) in β-O-4 structure
4.88	Protons on the side chain (Cβ) of benzene rings
4.97	Hydrogen proton of α site (Hα) in the β-O-4 structure
4.99–5.33	Protons in the structure of β-5
5.31	Hydrogen proton of α site (Hα) in the β-5 structure
5.71	β-vinyl proton in β-O-4, β-1 structure
6.05	Protons on the side chain (Cα) in the β-O-4, β-1 structure
6.10–8.00	Protons on aromatic rings
6.64–7.08	Protons on guaiacyl aromatic rings
7.25–7.62	Protons on *p*-hydroxyphenyl aromatic rings
8.00–11.50	Protons on carboxyl and aldehyde groups

The most important information shown in the ^1^H NMR spectra in Fig. 5 is the clear cut appearance of a new signal (3.12 ppm) at 60 °C LA-ChCl-L. Combined with the structure of ChCl (Fig. 1b) and the signal of nitromethyl in ^1^H NMR spectra (about 3.0 ppm), we can infer that the new signal was due to nitromethyl in ChCl with 9 equivalent hydrogens, and these results suggested that AL might be attached to ChCl when it dissolved in LA-ChCl at 60 °C. However, with the increase in the temperature or the addition of microwave, the connections became weaker, which indicates that the bonding strength between AL and ChCl was not strong, and that heat or microwave can break these connections.

**Fig. 6:**
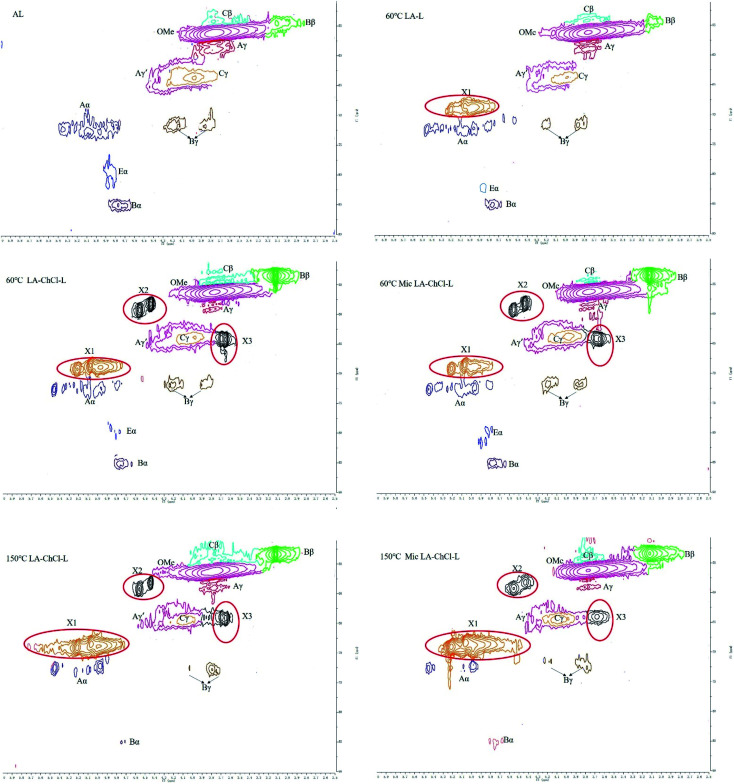
HSQC spectra of AL and various lignin treated on the condition of 60 °C, 60 °C with microwave, 150 °C and 150 °C with microwave, aliphatic regions (*δ*_C_/*δ*_H_ 50 to 90/2.5 to 6.0).

**Fig. 7:**
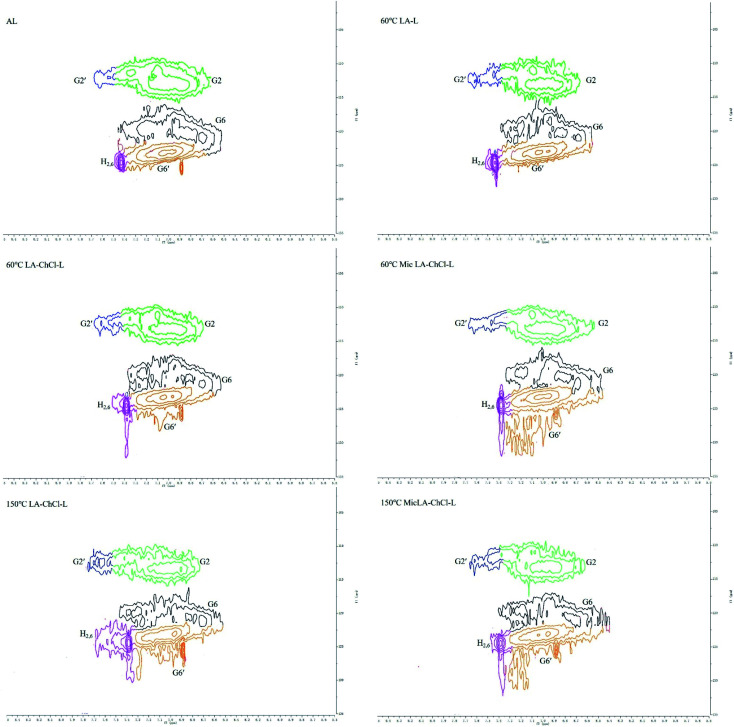
HSQC spectra of AL and various lignin treated on the condition of 60 °C, 60 °C with microwave, 150 °C and 150 °C with microwave, aromatic regions (*δ*_C_/*δ*_H_ 100 to 135/5.5 to 8.5).

**Fig. 8:**
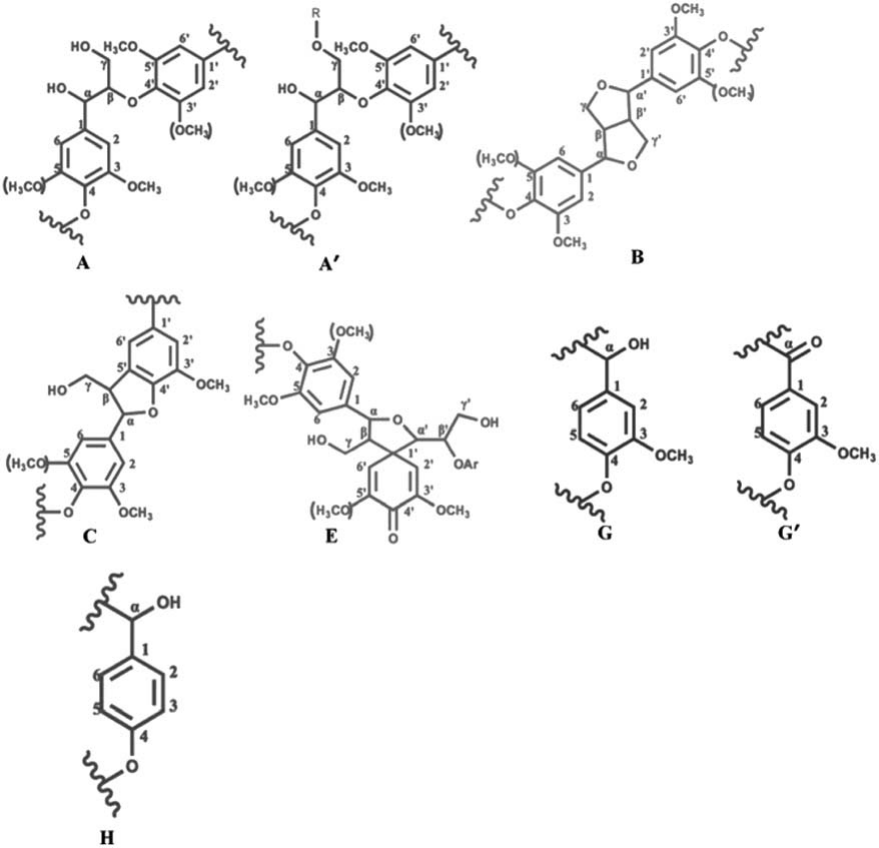
Main structure found in the lignin studied: (A) β-O-4′ aryl ether linkages with free –OH at the γ-carbon; (A′) β-O-4′ aryl ether linkages with acetylated and/or *p*-hydroxybenzoated-OH at γ-carbon; (B) resinol substructure formed by β–β′, α-O-β′ and γ-O-α′ linkages; (C) phenylcoumarane substructures formed by β-5′ and α-O-4 linkages; (E) spirodienone substructures formed by β-1′ and α-O-α′ linkages.

2D-HSQC NMR shows an essential structure characterization of the lignin, including unit linkages and unit types. Fig. 6 and 7 shows the spectra of studied lignin in aliphatic regions (*δ*_C_/*δ*_H_ 50 to 90/2.5 to 6.0) and aromatic regions (*δ*_C_/*δ*_H_ 100 to 135/5.5 to 8.5). The detailed cross signal assignments in the HSQC NMR spectra are shown in Table 6, and the substructures are observed in the HSQC NMR spectra in Fig. 8.

**Table 6:** Assignments of signals observed in the ^1^H/^13^C HSQC NMR spectra of lignin studied in this research

Labels	*δ*_C_/*δ*_H_ (ppm)	Assignment
MeO	56.23/3.77	C–H, methoxyls (MeO)
Aα	72.43/4.99	Cα–Hα, β-O-4′(A)
Aγ	58.49/3.79	Cγ–Hγ, β-O-4′(A)
Aγ′	63.11/4.33	Cγ–Hγ, of γ acylated β-O-4′(A)
Bα	85.25/4.77	Cα–Hα, resinol (B)
Bβ	53.48/3.13	Cβ–Hβ, resinol (B)
Bγ	(71.93/3.86) (71.86/4.20)	Cγ–Hγ, resinol (B)
Cβ	53.92/3.66	Cβ–Hβ, phenylcoumaran (C)
Cγ	63.83/3.98	Cγ–Hγ, phenylcoumaran (C)
Eα	80.12/4.88	Cα–Hα, spirodienone (E)
G2	111.9/6.99	C2–H2, G units (G)
G2′	112.69/7.61	C2–H2, oxidized (Cα=O) G units (G′)
G6	120.06/6.93	C6–H6, G units (G)
G6′	123.07/7.01	C6–H6, oxidized (Cα=O) G units (G′)
H2,6	124.69/7.43	C2,6–H2,6, *p*-hydroxyphenyl (H)

The HSQC NMR spectra of the aromatic regions in AL, 60 °C LA-L and 60 °C LA-ChCl-L are almost the same, indicating that the structure of lignin in aromatic regions was not influenced. The typical signals of the G and H unit appeared clearly, whereas the signal of S unit did not appear, which is in line with the FT-IR analysis and the feature of softwood.

In aliphatic regions, compared with AL, a new signal (X1, *δ*_C_/*δ*_H_ 69.08/5.20,68.54/5.07 and 68.84/4.97) appeared in 60 °C LA-L, which may belong to the C–H in LA. Moreover, it is interesting to note that two new signals (X2, *δ*_C_/*δ*_H_ 59.35/4.55 and 58.26/4.44; X3,64.01/3.69 and 64.13/3.65) appeared, which may belong to the C–H in ChCl, indicating that AL was attached by ChCl and LA when dissolved in LA-ChCl at 60 °C, which is in line with the ^1^H NMR analysis.

In addition, it also can be seen from Fig. 6 that the signal strength of the signal, which represented the β-O-4 bond (Aα, Aγ and Aγ′), became weaker in 60 °C LA-L and 60 °C LA-ChCl-L, particularly in 60 °C LA-ChCl-L, which indicated that the β-O-4 bond in 60 °C LA-L and 60 °C LA-ChCl-L was partly broken and that the lignin was depolymerized in the LA solvent, which was in line with the result of the GPC analysis for the decrease of *M*_w_.

**Fig. 9:**
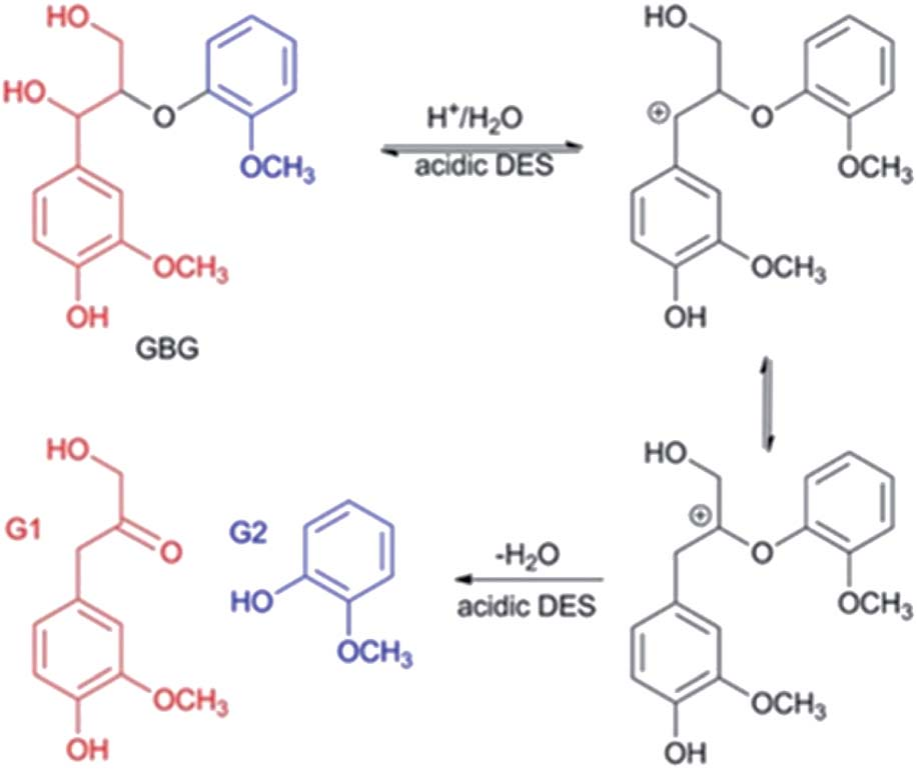
Possible mechanism of the selective cleavage of β-O-4 bond of lignin model compounds in acidic DES. GBG: guaiacylglycerol-*β*-guaiacyl ether.

**Fig. 10:**
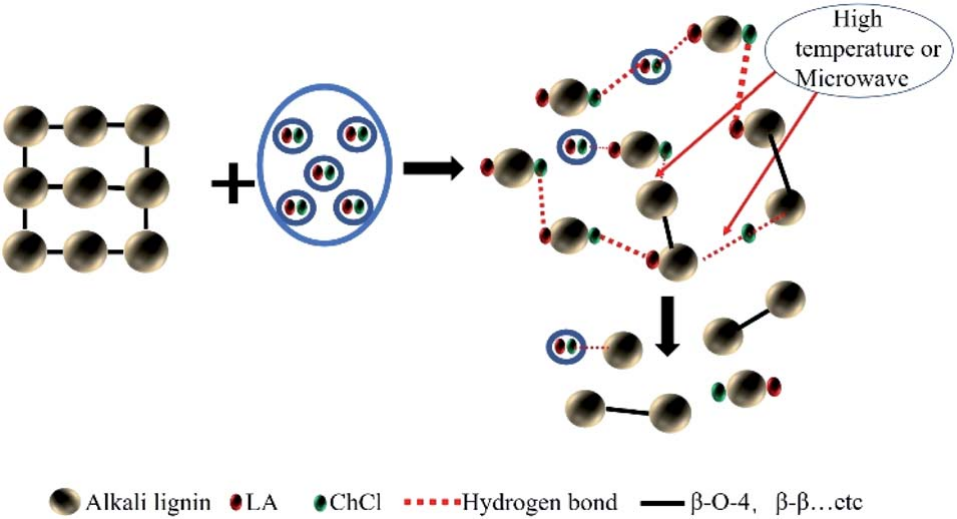
Possible dissolution mechanism of alkali lignin in LA-ChCl.

As for the 60 °C LA-ChCl-L, when AL was dissolved in LA-ChCl at 60 °C, it was depolymerized under the action of LA, thus leading to more cleavage of the β-O-4 bond in acidic DES (Fig. 9),^13^ and the ChCl in DES may have bonded with phenolic groups on the lignin,^13,27^ as previous researches reported. Therefore, the depolymerized lignin fragments would gather again under the action of ChCl, as shown in Fig. 10. This is a good explanation for the results of the GPC analysis that the average *M*_w_ of 60 °C LA-ChCl-L increased obviously, and the lignin content of small components decreased, while the whole *M*_w_ distribution became more concentrated.

#### Effect of high temperature and microwave on the structure of AL dissolved in LA-ChCl

It is well known that the structure of condensed lignin is stable and hard to destroy *via* heating or microwave. However, the connection between ChCl and phenolic groups on lignin may be vulnerable. In order to further confirm the reason for the increase in the *M*_w_ distribution in 60 °C LA-ChCl-L, the effect of high temperature and microwave was investigated.

It can be seen from the GPC analysis that the *M*_w_ distribution of 60 °C Mic LA-ChCl-L, 150 °C LA-ChCl-L and 150 °C Mic LA-ChCl-L decreased significantly compared with that of 60 °C LA-ChCl-L. The new signal (3.12 ppm) in ^1^H spectra, which appeared in the 60 °C LA-ChCl-L, appeared and became weaker in 60 °C Mic LA-ChCl-L, 150 °C LA-ChCl-L and 150 °C Mic LA-ChCl-L. As for the HSQC NMR analysis, the new signals (X2, X3) that appeared after the addition of ChCl, also appeared and became weaker in 60 °C Mic LA-ChCl-L, 150 °C LA-ChCl-L and 150 °C Mic LA-ChCl-L. All these results suggested that the connection between ChCl and phenolic groups on lignin was broken in parts, and the accumulated lignin fragments were redistributed under the action of heat and microwave, as shown in Fig. 10. In addition, the aromatic regions were nearly not influenced under the effect of high temperature and microwave based on the fact that all six lignin showed the same HSQC spectra in the aromatic regions.

## Conclusions

1. Compared with others tested and reported DESs, LA : ChCl in the ratio of 10 : 1 was an excellent solvent for AL, in which the solubility of AL was more than 17 wt% under a mild condition (60 °C).

2. By analyzing the structure of dissolved lignin, it was found that the AL was depolymerized due to the breakage of the β-O-4 bond, and the main structure of the aromatic regions of dissolved lignin was not changed.

3. The hydroxyl group of LA in DES could improve the polarity of the system and facilitate the depolymerization of lignin. ChCl could form a new connection with the dissolved lignin fragments, which made the molecular weight and homogeneity of lignin increase after dissolution and regeneration.

4. The bonding strength between ChCl and lignin was weak, which could be easily destroyed by using microwave and heating (150 °C).

## An expanded form of all the abbreviations

DESs: Deep eutectic solvents
AL: Alkali lignin
LA: Lactic acid
HBDs: Hydrogen bond donors
ChCl: Choline chloride
GPC: Gel permeation chromatography
FTIR: Fourier transform infrared spectrometer
^1^H and HSQC NMR: ^1^H and ^1^H–^13^C correlation two dimensional (2D) heteronuclear single-quantum coherence (HSQC) nuclear magnetic resonance (NMR)
LA-ChCl: Lactic acid : choline chloride 10 : 1(molar ratio)
HBAs: Hydrogen bond acceptors
DESL: Lignin isolated from DES
U: Urea
BA: Butanoic acid
60 °C LA-L: Lignin isolated from LA on the condition of 60 °C
60 °C LA-ChCl-L: Lignin isolated from LA-ChCl on the condition of 60 °C
60 °C Mic LA-ChCl-L: Lignin isolated from LA-ChCl on the condition of 60 °C with microwaves
150 °C LA-ChCl-L: Lignin isolated from LA-ChCl on the condition of 150 °C
150 °C Mic LA-ChCl-L: Lignin isolated from LA-ChCl on the condition of 150 °C with microwaves
*M*_w_
: Molecular weight
PD: Polydispersity

## Conflicts of interest

There are no conflicts of interest to declare

## Supplementary Material

RA-010-D0RA07808E-s001

RA-010-D0RA07808E-s002

RA-010-D0RA07808E-s003

RA-010-D0RA07808E-s004

RA-010-D0RA07808E-s005

RA-010-D0RA07808E-s006

RA-010-D0RA07808E-s007

RA-010-D0RA07808E-s008

RA-010-D0RA07808E-s009

RA-010-D0RA07808E-s010

RA-010-D0RA07808E-s011

RA-010-D0RA07808E-s012

RA-010-D0RA07808E-s013

RA-010-D0RA07808E-s014

RA-010-D0RA07808E-s015

RA-010-D0RA07808E-s016

RA-010-D0RA07808E-s017

RA-010-D0RA07808E-s018

RA-010-D0RA07808E-s019

RA-010-D0RA07808E-s020

RA-010-D0RA07808E-s021
